# Febrile Rhinovirus Illness During Pregnancy Is Associated With Low Birth Weight in Nepal

**DOI:** 10.1093/ofid/ofx073

**Published:** 2017-04-06

**Authors:** Erin K Philpott, Janet A Englund, Joanne Katz, James Tielsch, Subarna Khatry, Stephen C LeClerq, Laxman Shrestha, Jane Kuypers, Amalia S Magaret, Mark C Steinhoff, Helen Y Chu

**Affiliations:** 1 Department of Medicine, Seattle Children's Research Institute, Washington; 2 Department of Pediatrics, Seattle Children's Research Institute, Washington; 3 Department of Laboratory Medicine, Seattle Children's Research Institute, Washington; 4 Department of Biostatistics, Seattle Children's Research Institute, Washington; 5 Center for Clinical and Translational Research, Seattle Children's Research Institute, Washington; 6 Department of International Health, Johns Hopkins Bloomberg School of Public Health; 7 Department of Global Healthy, George Washington University, Washington DC; 8 Nepal Nutrition Intervention Project-Sarlahi, Nepal; 9 Department of Pediatrics, Institute of Medicine, Tribhuvan University Teaching Hospital, Kathmandu, Nepal; 10 Department of Global Health, Cincinnati Children's Hospital Medical Center, Ohio

**Keywords:** low birth weight, Nepal, pregnancy, preterm birth, rhinovirus

## Abstract

**Background:**

Adverse birth outcomes, including low birth weight (LBW), defined as <2500 grams, small-for-gestational-age (SGA), and prematurity, contribute to 60%–80% of infant mortality worldwide and may be related to infections during pregnancy. The aim of this study was to assess whether febrile human rhinovirus (HRV) illness is associated with adverse birth outcomes.

**Methods:**

Active household-based weekly surveillance was performed for respiratory illness episodes in pregnant women as part of a community-based, prospective, randomized trial of maternal influenza immunization in rural Nepal. Rhinovirus (HRV) febrile illness episodes were defined as fever plus cough, sore throat, runny nose, and/or myalgia with HRV detected on mid-nasal swab. Multivariate regression analysis evaluated the association between febrile HRV respiratory illness and adverse birth outcomes.

**Results:**

Overall, 96 (3%) of 3693 pregnant women had HRV-positive febrile respiratory illnesses. Infants born to pregnant women with HRV febrile illness had a 1.6-fold increased risk of being LBW compared with those with non-HRV febrile illness (28 of 96 [38%] vs 109 of 458 [24%]; relative risk [RR], 1.6; 95% confidence interval [CI], 1.1–2.3). No difference in risk of LBW was observed between infants born to mothers with non-HRV febrile respiratory illness and those without respiratory illness during pregnancy (109 of 458 [24%] vs 552 of 2220 [25%], respectively; RR, 1.0; 95% CI, 0.8–1.2).

**Conclusions:**

Febrile illness due to rhinovirus during pregnancy was associated with increased risk of LBW in a rural South Asian population. Interventions to reduce the burden of febrile respiratory illness due to rhinovirus during pregnancy may have a significant impact on LBW and subsequent infant mortality.

Adverse birth outcomes, including low birth weight (LBW), small-for-gestational age (SGA), and preterm birth (PTB), contribute to 60%–80% of infant mortality worldwide [1]. Infections during pregnancy impact risk of adverse birth outcomes and congenital anomalies [2, 3]. Respiratory viruses have been recognized as significant contributors to morbidity and mortality worldwide. Influenza during pregnancy, in particular avian influenza and pandemic H1N1, has been associated with spontaneous abortion, stillbirth, and prematurity [4, 5]. A prospective randomized trial in Bangladesh and an observational US-based study each found an association between influenza immunization during pregnancy and a reduction in SGA and an increase in mean birth weight [6, 7]. An impact on LBW and PTB was additionally noted in a meta-analysis of 5 randomized trials of maternal influenza immunization [8].

Human rhinovirus (HRV) is a respiratory virus first isolated in the 1950s and historically implicated as the etiology of the common cold [9, 10]. With improvements in molecular diagnostics, HRV has been increasingly recognized to cause serious illness requiring hospitalization and death among infants and young children, immunocompromised individuals, adults with chronic obstructive pulmonary disease, or asthma. Human rhinovirus has also been identified as the most common etiology of pneumonia in hospitalized adults [11–13]. However, the clinical presentation of HRV infection during pregnancy and effect on birth outcomes is not well established.

The risk of increased susceptibility and severity associated with certain infections during pregnancy is likely related to a complex interplay of the maternal-placental-fetal unit that is not well understood. It has been postulated that infection causes activation of the innate immune system stimulating the release of proinflammatory cytokines, which may underlie the pathogenesis of both PTB and LBW [14]. Identification of a relationship between HRV infection during pregnancy and adverse birth outcomes has the potential to drive improvement of diagnostics, infection control measures, and treatment options for HRV infection.

## METHODS

We conducted an analysis using samples and clinical data collected in a community-based, prospective placebo-controlled, randomized trial of maternal influenza immunization of 3693 pregnant women and their infants conducted in rural southern Nepal from 2011 to 2014. Methods of the parent study have been previously published [15]. In brief, illness episodes were identified through longitudinal household-based active weekly surveillance of women from the second trimester of pregnancy until 6 months postpartum. Mid-nasal swabs were collected from women meeting criteria for respiratory illness episode, which was defined as subjective fever plus 1 or more symptom (cough, sore throat, runny nose, or myalgia). Fever was included in the illness episode definition as part of the primary trial of maternal influenza immunization to capture influenza-like-illness during pregnancy [3]. Febrile HRV respiratory illness was defined as a respiratory illness episode with a nasal swab with HRV detected by real-time polymerase chain reaction (RT-PCR) assay. A unique illness episode was defined as an illness episode followed by 7 symptom-free days. Only the primary HRV illness episode was included in the analysis.

Adverse birth outcomes were defined as LBW (birth weight less than 2500 grams), PTB (birth at less than 37 weeks gestation), and SGA (birth weight <10 percentile of weight for gestational age). Gestational age was calculated based on last menstrual period according to a house-to-house census conducted every 5 weeks of all women of childbearing age [16]. If a woman had a missed period, then a urine pregnancy test was performed and she was enrolled in the study if her urine pregnancy test was positive. Therefore, the last menstrual period was used to date the infant’s gestational age in the study with a 5-week recall period. The frequencies of each adverse outcome were compared between infants born to mothers with febrile HRV respiratory illness during pregnancy and those without febrile HRV respiratory illness during pregnancy. Infant weights were included if they were obtained at less than 72 hours after birth. Small-for-gestational age was calculated based on the INTERGROWTH-21st criteria [17]. A composite adverse birth outcome was defined as an infant being preterm, LBW, and/or SGA.

Real-time PCR for detection of HRV was performed as described, with the modification of a degenerate C/T base at the 3’ end of the forward primer [18]. Real-time PCR for additional respiratory viruses, including respiratory syncytial virus, human metapneumovirus, parainfluenza viruses 1–4, influenza, coronavirus, bocavirus, and adenovirus, was performed using previously published methods [18–22]. Although the HRV RT-PCR assay has been shown to detect some enterovirus from culture lysates in which enterovirus was present in high copy numbers, this assay has not been positive when enterovirus-positive clinical respiratory specimens were tested as part of other studies and therefore less likely in this case to misdiagnose enterovirus as rhinovirus [23, 24]. Rhinovirus-positive samples collected from pregnant and postpartum women with cycle threshold values less than 30 were selected for sequencing using a previously published protocol [23]. For sequencing, previously extracted samples were used to generate complementary deoxyribonucleic acid using random hexamers, and sequencing was performed using primers targeting the 5’ noncoding region. Gel-extracted amplicons were sequenced (GeneWiz) and analyzed using Sequencher 4.1 software and compared with HRV reference sequences from GenBank using the nucleotide-nucleotide Basic Local Alignment Search Tool ([BLAST] www.blast.ncbi.nlm.nih.gov) algorithm. Trimmed sequences were aligned using Geneious R6.1 (Biomatters) and Seaview 4.4 [25]. Phylogenetic reconstruction was performed using the maximum likelihood method with 100 bootstrap replicates using PhyML 3.0 within DIVEIN [26]. Neighbor joining trees were drawn and edited using FigTree v1.4.1. Sequences were considered to be divergent genotypes if they differed from other study sequences by >2% when compared using BLAST [23]. Sequence data were not of sufficient length to be submitted to GenBank but are available upon request.

Log-linear regression was used to assess the association of baseline characteristics of pregnant women with the incidence of HRV respiratory illness during pregnancy; person-years (p-y) at risk of infection was included as an exposure. Adverse birth outcomes such as LBW, SGA, and PTB were also assessed using log-linear regression. Potential risk factors analyzed include (1) maternal febrile respiratory illness during pregnancy and, simultaneously, (2) a specific history of HRV-associated respiratory illness. For the outcomes of LBW, we also included a term indicating HRV-associated respiratory illness in the second trimester, to assess whether timing during pregnancy was influential. To avoid confounding, additional covariates included season of birth and infant sex in the analysis. The Wilcoxon-rank sum statistic was used to compare symptom duration between pregnant and postpartum women with febrile HRV as well as pregnant women who gave birth to LBW infants compared with those who did not.

Institutional review board (IRB) approval for the parent study was obtained from the Johns Hopkins University Bloomberg School of Public Health, Seattle Children’s Hospital, Cincinnati Children’s Hospital, the Institute of Medicine at Tribhuvan University, and the Nepal Health Research Council. Oral consent was obtained from study participants due to low literacy rates in the population. This procedure was approved by the IRB. The trial in which this substudy was conducted is registered at Clinicaltrials.gov (NCT01034254).

## RESULTS

A total of 191 (5%) of 3693 women had a febrile HRV respiratory illness episode, 96 (50%) of which occurred during pregnancy and 95 (50%) occurred after delivery. The median person-weeks (p-w) of follow up was 51 weeks (interquartile range [IQR], 46–56) for febrile HRV respiratory illness during pregnancy and 49 weeks (IQR, 43–55) for those without HRV-associated illness while pregnant. Febrile HRV respiratory illness incidence was 58.3/1000 p-y. The incidence of febrile HRV illness while pregnant was 95 of 1497 p-y, or 64.1/1000 p-y, and incidence after delivery was 95 of 1799 p-y, or 52.8/1000 p-y (*P* = .51). Characteristics of women with and without febrile HRV respiratory illness during pregnancy and their infants are outlined in Table 1. The median gestational age at infection among the febrile HRV respiratory illness group was 28 weeks (IQR, 12–39). Incidence of HRV was similar by baseline characteristics, including maternal age, maternal literacy, nulliparity, indoor biomass cookstove use, and number of children in the household. In particular, no difference in maternal body mass index was observed. In addition, no difference was observed in influenza vaccination status between the 2 groups. Other viruses detected in association with febrile respiratory illness episodes included respiratory syncytial virus, human metapneumovirus, parainfluenza viruses 1–4, influenza, coronavirus, bocavirus, and adenovirus each occurring at notably lower frequency compared with HRV.

**Table 1.** Baseline Characteristics Based on Maternal Illness Status During Pregnancy

Characteristic (Median [IQR] or No., %)	HRV-Positive Illness (*n* = 96)	No HRV Illness (*n* = 3597)	RR (CI)	*P* Value
Maternal age at enrollment, years	23 (20–26)	23 (20–26)	0.99 (0.88–1.12)	.93
Flu vaccination during pregnancy	50 (52%)	1808 (50%)	1.06 (0.37–3.10)	.91
Maternal body mass index at enrollment	20.5 (18.8–22.4)	20.7 (19.0–22.6)	0.97 (0.81–1.17)	.77
Maternal education, years	3 (0–9)	5 (0–10)	0.97 (0.86–1.09)	.61
Maternal literacy	49 (57.6%)	1967 (60.4%)	0.90 (0.28–2.85)	.86
Gestational age at enrollment, weeks	18 (12–21)	17 (13–23)	0.98 (0.91–1.07)	.69
First pregnancy	38 (39.6%)	1510 (42.1%)	0.92 (0.31–2.74)	.88
Gestational age at infection, weeks	28 (22–33)	-		
Smoking at enrollment	2 (2.4%)	105 (3.2%)	0.76 (0.02–30.67)	.88
Madeshi ethnic group	45 (49.5%)	1482 (42.8%)	1.30 (0.43–3.92)	.65
Brahmin caste	11 (12.1%)	369 (10.7%)	1.17 (0.22–6.23)	.86
Protected water source	11 (12.1%)	525 (15.2%)	0.77 (0.14–4.15)	.76
Presence of in-home latrine	38 (41.8%)	1709 (49.4%)	0.74 (0.24–2.25)	.59
In-home electricity	84 (92.3%)	3112 (89.9%)	1.28 (0.17–9.90)	.81
Presence of indoor cookstove	80 (87.9%)	2815 (81.2%)	1.68 (0.31–9.10)	.55
Biogas, gas, kerosene fuel used	6 (6.6%)	330 (9.5%)	0.66 (0.07–5.99)	.71
No. of children <5 in household	1 (0–1)	0 (0–1)	1.07 (0.55–2.07)	.84
No. of children <15 in household	1 (1–3)	2 (1–3)	0.96 (0.68–1.35)	.81
Number household members	7 (4–10)	7 (5–10)	0.99 (0.85–1.14)	.87
Persons per room in household	3 (2–4)	3 (2–4)	0.97 (0.77–1.23)	.82
Male sex of infant	56 (58.3%)	1881 (52.3%)	1.26 (0.43–3.71)	.68
Infant alive at birth	95 (99%)	3526 (98.3%)	1.57 (0.01–295.74)	.87

Abbreviations: CI, confidence interval; HRV, human rhinovirus; IQR, interquartile range; RR, relative risk.

Seventy-four of 96 (77.1%) infants born to pregnant women with febrile HRV respiratory illness and 2678 of 3597 (74.5%) infants born to mothers without HRV-associated illness were weighed within 72 hours of birth and therefore included in the assessment of LBW and SGA. Of 2752 infants with birth weight measured in the first 72 hours, 84% (2302) were taken on the day of birth. Median weight in those measured on days 2 and 3 of life was approximately 0.1 kg lower than those measured on day 1 (2.73 at day 2 and 2.69 at day 3 vs 2.80 on day 1), and the proportion determined to be of LBW increased from 24% on day 1 to 27% on day 2 to 36% on day 3. Had all 3 days observed exactly the same rate of LBW designations of 24%, it is expected then that approximately 27 infants (9 weighed on day 2 and 18 weighed on day 3 of life) would not have been determined to have LBW. With 689 infants classified as LBW, however, 27 is only 4% of that group, so this comprises a very low potential misclassification rate.

**Figure 1.**
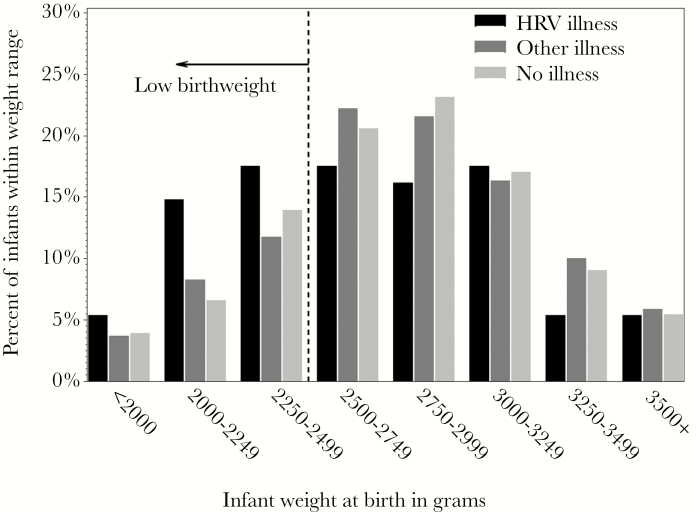
Frequency of low birth weight based on maternal respiratory illness status during pregnancy. Bar plot comparing pregnant women with febrile rhinovirus respiratory illness (black) vs pregnant women with febrile respiratory illness due to another respiratory virus (dark gray) vs pregnant women without febrile respiratory illness during pregnancy (light gray). HRV, human rhinovirus.

Prevalence of LBW was significantly higher among infants born to pregnant women with febrile HRV respiratory illness compared with those born to mothers with a febrile respiratory illness of another viral etiology during pregnancy, after adjusting for season of birth and gender of the infant, with a 1.6-fold increased risk of LBW (28 of 74 [38%] vs 109 of 458 [24%], respectively; relative risk [RR], 1.6; 95% confidence interval [CI], 1.1–2.3) (Figure 1; Table 2). When comparing pregnant women with non-HRV febrile viral respiratory illness to those with no febrile respiratory illness, no difference in risk of LBW was observed (109 of 458 [24%] vs 552 of 2220 [25%], respectively; RR, 1.0; 95% CI, 0.8–1.2). Mean birth weight was 2.58 kg (2.34–3.01) in the group of infants exposed to maternal febrile HRV illness compared with 2.80 kg (2.50–3.08) among infants not exposed to respiratory illness during pregnancy.

**Table 2.** Prevalence of Adverse Birth Outcomes Based on Maternal Illness Status During Pregnancy

	Respiratory Illness Status During Pregnancy^a^						
Adverse Birth Outcome, No. (%)	HRV^+^ Illness (*n* = 96)	Non-HRV Illness (*n* = 597)	No Illness (*n* = 3000)	RR (95% CI)^c^ For Illness	*P* Value	RR (95% CI)^d^ For HRV^+^ Illness	*P* Value
Birth weight (median, IQR)^a^	2.58 (2.34–3.01)	2.78 (2.52–3.09)	2.80 (2.50–3.07)				
Low birth weight (*n* = 2752)^b^	28 (37.8%)	109 (23.8%)	552 (24.9%)	1.0 (0.8–1.2)	.60	1.6 (1.1–2.3)	.0079
Preterm (*n* = 3644)	9 (9.5%)	91 (15.5%)	382 (12.9%)	1.2 (1.0–1.5)	.075	0.6 (0.3–1.1)	.12
Very preterm^e^ (*n* = 3644)	1 (1.1%)	15 (2.6%)	49 (1.6%)	1.6 (0.9–2.8)	.13	0.4 (0.1–3.1)	.39
Gestational age (median, IQR)	40 (38–41)	39 (38–41)	40 (38–41)				
Small for gestational age (*n* = 2751)	34 (46.0%)	176 (38.4%)	839 (37.8%)	1.0 (0.9–1.2)	.85	1.2 (0.9–1.6)	.20
Composite adverse birth outcome (*n* = 2889)	44 (57.9%)	266 (54.9%)	1208 (51.9%)	1.1 (0.9–1.2)	.24	1.1 (0.8–1.3)	.62

Abbreviations: CI, confidence interval; HRV, human rhinovirus; IQR, interquartile range; RR, relative risk.

^a^Relative risk calculated using multivariable Poisson regression for each outcome with 4 predictors, including (1) an indicator for any febrile illness (1 = HRV febrile illness, or other febrile illness, 0 = no febrile illness) and (2) another indicator for HRV (1 = HRV, 0 = no HRV), as well as (3) season of birth (fall versus other) and (4) gender of infant.

^b^Infants weighed within 72 hours were included in the low birth weight analysis.

^c^Relative risk for febrile illness is the increased risk of having the adverse outcome given any febrile illness.

^d^Relative risk for HRV is the increased risk of having the adverse outcome if the febrile illness additionally involved HRV versus no HRV (but could include other febrile illness).

^e^Very preterm is defined as birth <34 weeks gestation.

**Figure 2.**
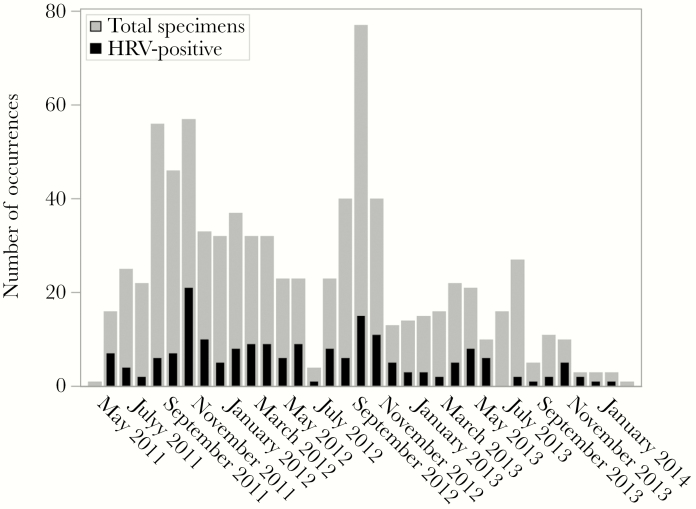
Seasonality of febrile respiratory illness among pregnant and postpartum women. Bar graph showing month of year (x-axis) and number of febrile respiratory illness episodes in pregnant and postpartum women (y-axis). Light gray represents total febrile respiratory illness episodes, and black represents febrile rhinovirus respiratory illness episodes. HRV, human rhinovirus.

Three HRV infections occurred in the first trimester of pregnancy, 40 in the second and 53 in the third. The lack of HRV infection in the first trimester is due to enrollment of women into the study beginning at 17 weeks gestation or later rather than lack of rhinovirus infection during the first trimester of pregnancy. Low birth weight prevalence was not affected by the trimester of pregnancy in which the HRV infection occurred (*P* = .91). Febrile HRV respiratory infection occurred year-round with a peak in October (Figure 2) and accounted for a consistent 5% of respiratory virus infections each month. Low birth weight occurred at a monthly rate of 20%–30% (Supplementary Figure 2). Due to the nature of enrollment, the number of patients under surveillance and therefore the numbers of samples collected were not constant over time, and this is reflected in the numbers of proportions of births and specimens tested (Figure 2 and Supplementary Figure 2).

Small-for-gestational age appeared higher among infants born to mothers with febrile HRV respiratory illness versus those with non-HRV febrile respiratory illness, although this did not reach statistical significance (34 of 74 [46.0%] vs 176 of 458 [38.4%]; *P* = .20). No significant difference in preterm delivery was found between the infants born to mothers with febrile HRV respiratory illness compared with no respiratory illness (9 of 95 [9.5%] vs 382 of 2961 [12.9%]; *P* = .12); although there was a trend toward a higher rate of PTBs among mothers with non-HRV respiratory illness compared with those without respiratory illness (91 of 588 [15.5%] vs 382 of 2961 [12.9%]; RR, 1.2; 95% CI, 1.0–1.5; *P* = .075).

Illness and symptom duration subdivided by LBW and pregnancy status are outlined in Table 3. The median duration of illness was 5 days (range, 1–49) among pregnant and postpartum women. No difference between overall illness duration was observed when comparing women with febrile HRV respiratory infection with and without LBW infants or when comparing women with febrile HRV respiratory illness during pregnancy versus after delivery. Six women died during the period of surveillance, none of whom had febrile HRV respiratory infection. Thirty-seven of 96 (39%) women with febrile HRV respiratory illness during pregnancy sought medical care. Overall, 7 women sought care from a physician or a hospital. Among women with febrile HRV respiratory infection during pregnancy, care-seeking behavior was similar between women who had LBW infants and those who did not (10 of 28 [35.7%] vs 20 of 46 [43.5%]; *P* = .51). In this same group with febrile HRV respiratory illness during pregnancy, the proportion seeking the care of a physician or hospital was also similar by LBW status of the infant. Three of 28 (10.7%) who had LBW infants saw a physician, compared with 4 of 46 (43.5%) who did not (*P* = .77). Of the 3 women with LBW infants who sought care from a physician, 1 woman sought care for severe headache and fever, whereas the reason for seeking care from a physician for the 2 other women was not documented.

**Table 3.** Analysis of Clinical Symptom Duration by Pregnancy Status and Low Birth Weight Status Women With Febrile HRV Respiratory Illness

Symptom Duration Median (Range)	Low Birth Weight Status in Pregnant Women With Rhinovirus			Pregnancy Status in Women With Rhinovirus		
	With LBW (*n* = 28)	Without LBW (*n* = 46)	*P* Value	Pregnant	Postpartum	*P* Value^a^
Any symptom	5 (1–17)	5 (1–49)	.99	5 (1–49)	5 (1–38)	.71
Fever	3 (1–7)	2 (1–10)	.15	2 (1–10)	2 (1–16)	.042^b^
Cough	3 (0–17)	3 (0–13)	.91	3 (0–25)	3 (0–21)	.63
Sore throat	2 (0–17)	2 (0–10)	.75	2 (0–17)	0 (0–14)	.018^c^
Runny nose	1 (0–17)	2 (0–7)	.82	2 (0–38)	3 (0–28)	.49
Myalgia	2 (0–14)	0 (0–49)	.33	0 (0–49)	0 (0–38)	.66

Abbreviations: HRV, human rhinovirus; LBW, low birth weight.

^a^Wilcoxon-rank sum was used to compare symptom duration between pregnant and postpartum women with febrile HRV.

^b^Significantly longer duration when postpartum versus pregnant.

^c^Significantly longer duration when pregnant versus postpartum.

Of the 191 HRV-positive samples, 41 (21.5%) of the samples had computed tomography values of <30 and were therefore successfully sequenced (Supplementary Figure 1). Of these, 27 (65.8%) were from pregnant women, whereas the remainder were from women who had rhinovirus after giving birth. Species A (24 of 27 [89%]) accounted for a majority of the samples sequenced, whereas Species B and C accounted for 2 of 27 (7.4%) and 1 of 27 (3.7%), respectively. Among women who gave birth to LBW infants, 6 of 7 (86%) were Species A and 1 of 7 (14%) were Species B. However, the proportion of samples sequenced compared with the total was small.

Eleven (11%) pregnant women with HRV had an additional respiratory virus detected including coronavirus (*n* = 2), human metapneumovirus (*n* = 5), adenovirus (*n* = 1), and bocavirus (*n* = 2), and respiratory syncytial virus and bocavirus (each, *n* = 1). Coinfections occurred at equal frequencies among mothers with and without LBW infants (3 of 28 [11%] vs 6 of 46 [13%], respectively; *P* = .77). The association between LBW and febrile HRV-illness episode remained when infants born to mothers with febrile HRV with viral coinfection illness episodes were excluded from the analysis (RR, 1.7; 95% CI, 1.1–2.4; *P* = .0089). No HRV-associated respiratory illness was found in association with influenza coinfection.

## DISCUSSION

Over a 3-year period of prospective active home-based weekly surveillance in a rural subtropical setting, we characterize the incidence of febrile respiratory illness due to rhinovirus in pregnant women. Limited data exist examining the relationship between HRV infection during pregnancy and adverse birth outcomes. In this study, we found that pregnant women with a febrile HRV respiratory illness were at 1.6-fold increased risk to have a LBW infant, compared with pregnant women without HRV illness.

Several aspects of the study design allowed for an accurate assessment of the impact of febrile rhinovirus illness during pregnancy on adverse birth outcomes. The study was conducted in a region of the world where PTB and LBW are common. In addition, in a setting with limited antenatal care and frequent home births, birth weight was measured within 72 hours of birth in the majority of infants. Dating of gestational age was performed using last menstrual period obtained through 5-weekly surveillance for pregnancy in women of childbearing age, leading to more accurate dating of gestational age compared with recall of last menstrual period at time of birth. Finally, intensive active weekly home-based respiratory illness surveillance allowed for collection of daily symptom data, and specimen collection in temperature-stable buffer permitted detection of rhinovirus and other respiratory viruses by sensitive molecular assays.

Similar to other subtropical regions, we found that febrile HRV respiratory infection occurred year-round with a peak in October and a nadir in June. Low birth weight has been shown to vary by season due to factors such as food insecurity [27]; however, we observed a relatively steady rate of LBW among infants born to women without febrile HRV respiratory illness ranging from 20% to 30% without significant fluctuations.

Pregnant women are at high risk for severe complications due to influenza, with increased rates of morbidity, mortality, and adverse fetal outcomes most clearly demonstrated with 2009 pandemic H1N1 [28]. Previous studies, including a study in our region, showed that febrile respiratory infection due to RSV was rare during pregnancy [29]. Limited data exist regarding the clinical presentation and severity of rhinovirus illness among pregnant women. Human rhinovirus accounts for 18%–26% of cases of viral pneumonia in hospitalized children and was detected in approximately half of children admitted to the intensive care unit for lower respiratory tract infection [30, 31]. In adults, HRV is the most frequent cause of community-acquired pneumonia requiring hospitalization in the United States [12, 32, 33]. We find that 39% of women with rhinovirus infection sought care for their illness, and that 7% of these were seen by a physician or at a hospital. This compares to an international study of community-based elderly adults, where 15% of those with rhinovirus illness were hospitalized [34].

The relationship between febrile rhinovirus infection during pregnancy and LBW has not been well studied. Low birth weight is the result of either preterm delivery or poor growth of the fetus resulting in intrauterine growth restriction (IUGR). Rates of PTB did not differ in women with and without febrile HRV respiratory illness during pregnancy, suggesting that IUGR was the primary mechanism leading to LBW associated with HRV during pregnancy. We did not find a significant effect of febrile rhinovirus illness during pregnancy on PTBs or SGAs. We note that approximately half (52%, or 719 of 1380) of infants with any poor outcomes are affected by only 1 of 3 adverse birth outcomes (LBW, PTB, or SGA); and only 41 (1%) of infants are affected by all 3 adverse birth outcomes. Of 688 infants with LBW, 76% are also SGA (compared with 25% of 2063 who are not LBW but are SGA). Likewise, of 688 with LBW, 26% are also preterm (compared with only 8% of 2063 who are not LBW but are preterm). Therefore, although there is evident correlation between these outcomes, they are not completely overlapping. Therefore, these outcomes are defining distinct populations and, in our study, may demonstrate different associations with risk factors.

Factors associated with IUGR, including low socioeconomic status, literacy, parity, poor nutritional status, smoking, and alcohol use, were similar between the groups with and without rhinovirus in our study [35]. Nepal has one of the highest rates of malnutrition in South Asia, with up to 41% of the general population having evidence of undernourishment [36]. More importantly, in our study, pregnant women with and without HRV-associated respiratory illness had similar mean body mass indices of 21 at the time of enrollment, suggesting that poor nutritional status associated with chronic illness is unlikely to be the etiology of the association with LBW.

We also note that febrile illness during pregnancy alone was not associated with an increased risk of LBW in our study. Of mothers with febrile non-HRV respiratory illness, only 24% of the infants were LBW, which is similar to the 25% without febrile respiratory illness during pregnancy. Fever is associated with increased circulation of cytokines, in particular interleukin-8, and has been postulated to be part of the reason why women with influenza may be at increased risk of severe disease and adverse birth outcomes [3]. We did not find an effect of febrile respiratory illness without the detection of rhinovirus in this study, however, making this less likely to be responsible for the birth weight effect seen in this study.

The birth weight effect in our study additionally does not appear to be due to increased duration or severity of illness in pregnant women with LBW infants. Prolonged illness during pregnancy may be associated with decreased intake and poor weight gain. Poor weight gain in pregnancy has been associated with IUGR [37]. We find that pregnant women with rhinovirus had a median 5 days of symptoms, and that this did not differ in those with LBW or in those who had rhinovirus during pregnancy or after birth. No significant difference in hospitalizations, physician visits, or other care seeking was observed among pregnant women with febrile HRV illness who did and did not give birth to LBW infants. Because of the potential for flu vaccination to reduce risk of LBW or PTBs, we evaluated for a difference in the incidence of rhinovirus febrile illness in women who did and did not receive the flu vaccine during pregnancy. We found no difference with flu vaccination. In addition, no mothers with rhinovirus febrile illness had evidence of influenza coinfection as a potential etiology of LBW.

Limitations of our study include our lack of active surveillance for respiratory viruses among asymptomatic or afebrile women, thereby limiting our ability to discern whether LBW was more strongly associated with HRV specifically or with any respiratory illness episode and its associated inflammation. Use of subjective fever as criteria for respiratory illness rather than use of a measured temperature may have also decreased our ability to detect respiratory illnesses. We also did not conduct surveillance before 17 weeks gestation, and, therefore, it is possible that some women had infections earlier in pregnancy that we were not able to identify in our study. Gestational age dating was limited by lack of first-trimester ultrasound; however, 5-weekly prospective home surveillance minimized maternal recall error of last menstrual periods. Illness episode severity measures, such as radiographic evidence of pneumonia or use of supplemental oxygen, were not evaluated because medical records of healthcare visits were not reviewed in this study. We were also unable to assess medical comorbidities in the participants, given the lack of access to primary or antenatal care in the study region. Other limitations include lack of detection of bacterial pathogens in this study. Human rhinovirus infections have been shown to augment respiratory bacterial colonization and infection; therefore, it is possible that bacterial infections may be contributing to the described febrile illnesses and play a role in the relationship with LBW. Mid-nasal samples were only obtained once weekly, and brief episodes of viral shedding, as commonly observed in adults with pre-existing immunity, could have been missed. The association of virologic factors, such as viral load or subtype, with disease severity, were also unable to be assessed due to a lack of a method to quantify rhinovirus by our RT-PCR assay and our limited ability to sequence the majority of the isolates. The study was performed within a single geographic locale with specific demographics and risk factors, compromising generalizability. Finally, because this is an observational study, we were unable to assess for a causal association between rhinovirus febrile respiratory illness and LBW. The groups with and without rhinovirus infection were similar in baseline sociodemographics, including factors that may affect LBW or risk of respiratory viral infections, such as maternal parity, maternal body-mass index, maternal literacy, household smoking, household density, and running water.

## CONCLUSIONS

In conclusion, we demonstrate that HRV is the most common cause of febrile respiratory illness in pregnant women in rural southern Nepal during our study period, and that HRV during pregnancy was associated with an increased risk of LBW. Because LBW infants have an increased risk of mortality compared with their heavier counterparts and HRV is a highly prevalent respiratory virus, this may represent a potential modifiable risk factor to reduce risk of LBW infants, particularly in developing countries. Interventions to reduce the burden of febrile respiratory illness due to rhinovirus during pregnancy, such as measures to improve infection control, development of targeted therapies, or improvement of diagnostics, may have a significant impact on LBW and subsequent infant mortality worldwide.

## Supplementary Data

Supplementary materials are available at *Open Forum Infectious Diseases* online. Consisting of data provided by the authors to benefit the reader, the posted materials are not copyedited and are the sole responsibility of the authors, so questions or comments should be addressed to the corresponding author.

## Supplementary Material

ofx073_suppl_1700065_supplemental_figure_1

ofx073_suppl_1700065_supplemental_figure_2

## Notes

Presented in part: Infectious Disease Society of America Annual Meeting (October 2016), New Orleans, LA.
