# Erratum

**DOI:** 10.1093/ofid/ofx182

**Published:** 2018-01-30

“Febrile Rhinovirus Illness During Pregnancy Is Associated With Low Birth Weight in Nepal” by Philpott et al. [Open Forum Infect Dis **2017**; 4(2)] is an Open Access article and should have contained the following copyright line: © The Author [2018]. Published by Oxford University Press [on behalf of

Infectious Diseases Society of America]. This is an Open Access article distributed under the terms of the Creative Commons Attribution License (http://creativecommons.org/licenses/by/4.0/), which permits unrestricted reuse, distribution, and reproduction in any medium, provided the original work is properly cited.

The authors regret these error.

