# Supplementary figures and images for: Febrile Rhinovirus Illness During Pregnancy Is Associated With Low Birth Weight in Nepal
Source: Open Forum Infect Dis. 2017 Apr 6;4(2):ofx073. doi: 10.1093/ofid/ofx073 (PMC5450902; doi:10.1093/ofid/ofx073)

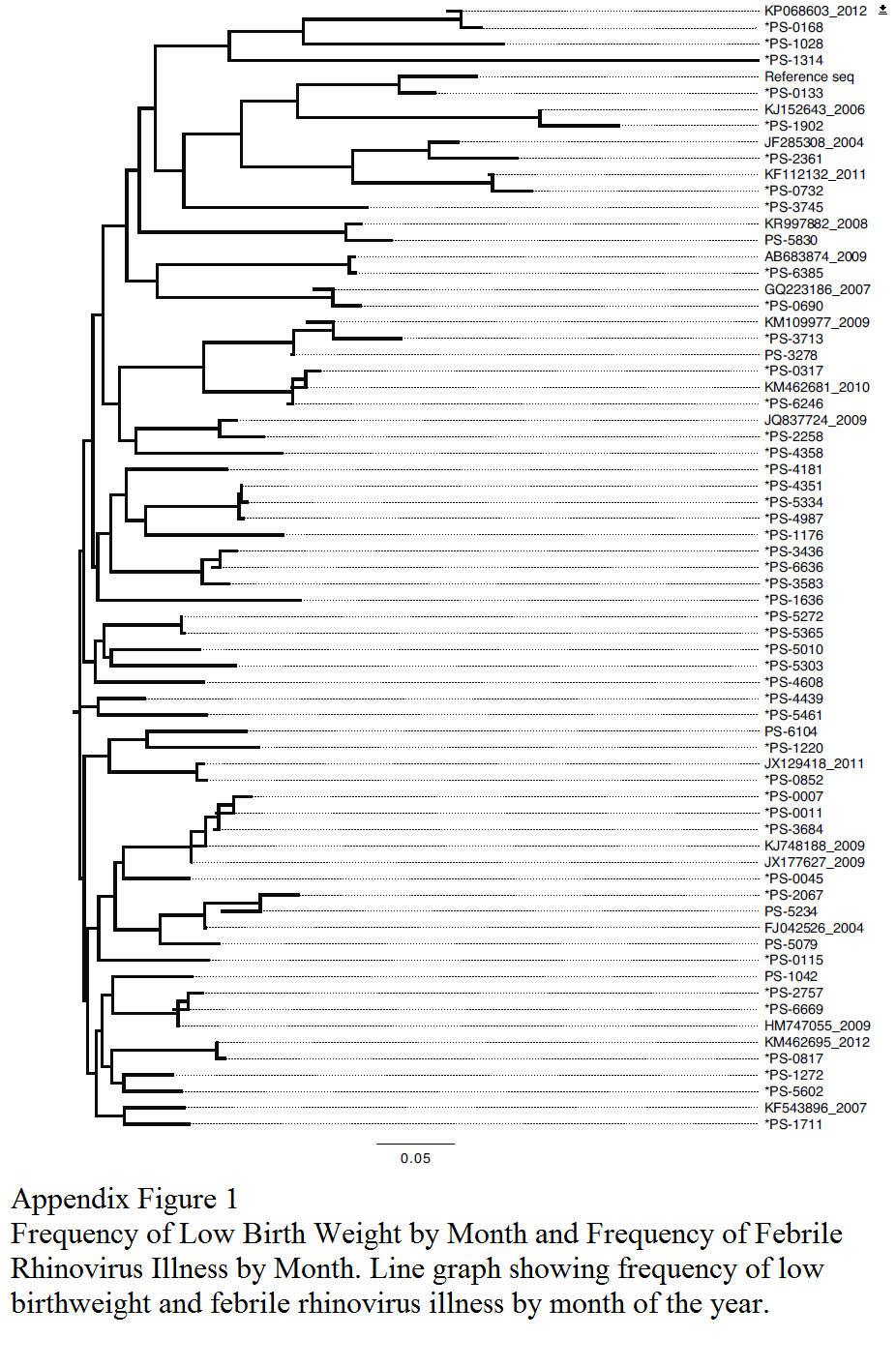

Supplement: ofx073_suppl_1700065_supplemental_figure_1 [file ofx073_suppl_1700065_supplemental_figure_1.png]

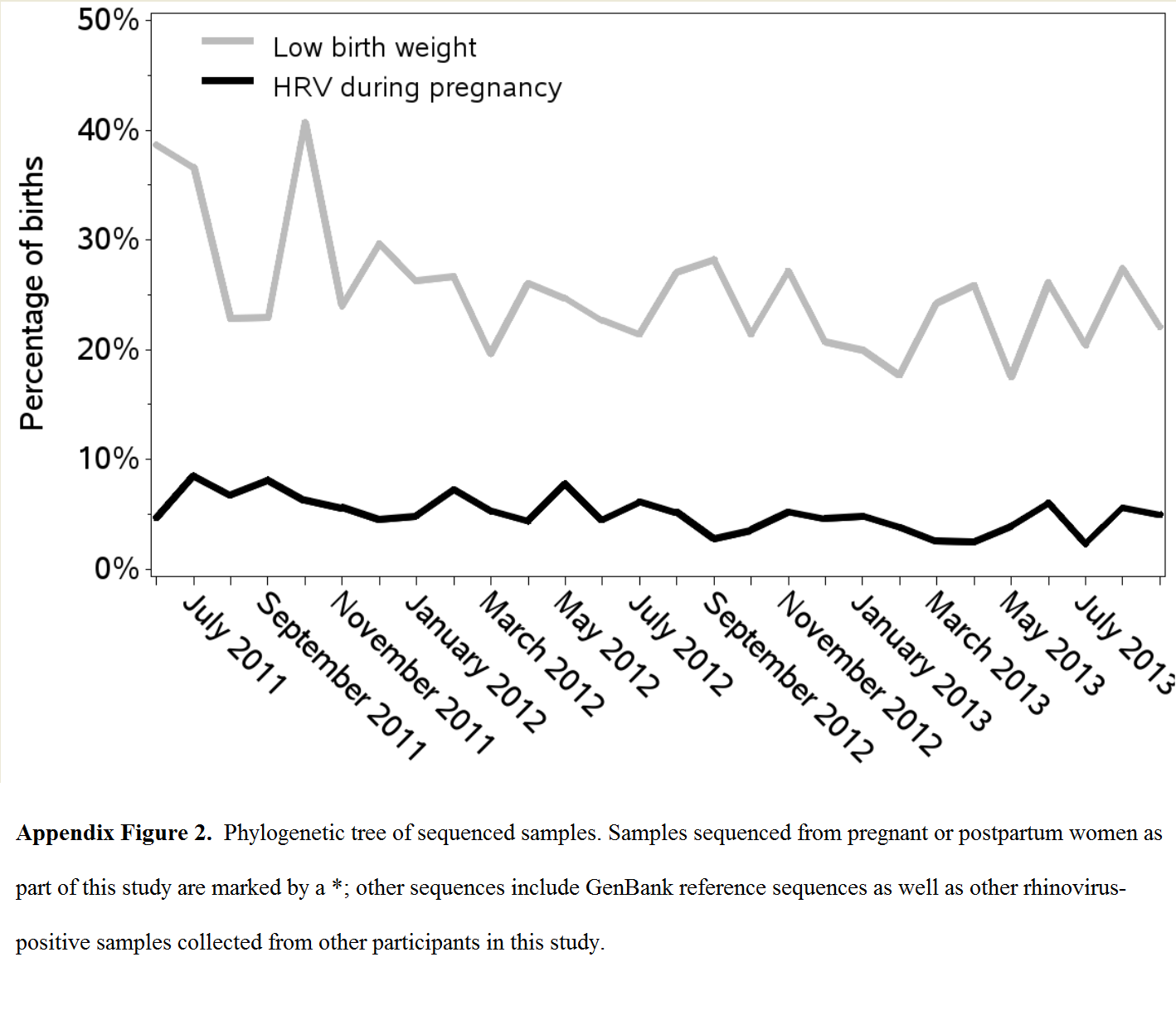

Supplement: ofx073_suppl_1700065_supplemental_figure_2 [file ofx073_suppl_1700065_supplemental_figure_2.png]
